# One size does not fit all: older adults benefit from redundant text in multimedia instruction

**DOI:** 10.3389/fpsyg.2015.01076

**Published:** 2015-07-28

**Authors:** Barbara Fenesi, Susan Vandermorris, Joseph A. Kim, David I. Shore, Jennifer J. Heisz

**Affiliations:** ^1^Applied Cognition in Education Lab, Department of Psychology, Neuroscience & Behaviour, McMaster University, HamiltonON, Canada; ^2^Neuropsychology and Cognitive Health Program, Baycrest Centre for Geriatric Care, TorontoON, Canada; ^3^Multisensory Perception Lab, Department of Psychology, Neuroscience & Behaviour, McMaster University, HamiltonON, Canada; ^4^Department of Kinesiology, McMaster University, HamiltonON, Canada

**Keywords:** aging, cognition, multimedia, instruction, learning

## Abstract

The multimedia design of presentations typically ignores that younger and older adults have varying cognitive strengths and weaknesses. We examined whether differential instructional design may enhance learning in these populations. Younger and older participants viewed one of three computer-based presentations: Audio only (narration), Redundant (audio narration with redundant text), or Complementary (audio narration with non-redundant text and images). Younger participants learned better when audio narration was paired with relevant images compared to when audio narration was paired with redundant text. However, older participants learned best when audio narration was paired with redundant text. Younger adults, who presumably have a higher working memory capacity (WMC), appear to benefit more from complementary information that may drive deeper conceptual processing. In contrast, older adults learn better from presentations that support redundant coding across modalities, which may help mitigate the effects of age-related decline in WMC. Additionally, several misconceptions of design quality appeared across age groups: both younger and older participants positively rated less effective designs. Findings suggest that one-size does not fit all, with older adults requiring unique multimedia design tailored to their cognitive abilities for effective learning.

## Introduction

Optimal learning through multimedia design requires a careful combination of words and images. Most research examining the factors promoting optimal multimedia learning has focused on young adults, with little known about the factors promoting optimal multimedia learning in older adults. Previous work suggests that there may be age-dependent differences; for example, when considering the optimal presentation of news media, one study found that older adults retained most information when presented through narration alone whereas younger adults benefited the most when narration was paired with either written text or video imagery (Stine et al., 1990). Importantly, the rapid rise of online courses in higher education, and an increasingly technology-oriented education system drives the need for practical research to address effective multimedia design across diverse age groups. Some researchers suggest that existing principles of instructional design can be used to accommodate the needs of older learners (Van Gerven et al., 2006). This is because existing instructional theories bear important benefits for older learners in that they support an efficient use of available cognitive resources. However, age-related decline in working memory capacity (WMC; Hedden and Gabrieli, 2004; Mattay et al., 2006) and processing resources (Pachman, 2007; Pachman and Ke, 2012) may mean that older adults require different design features than younger adults for optimal learning. Indeed, some argue that design for older adults should involve understanding their unique capabilities and limitations, identifying their needs, preferences and desires for technology in their lives, and involving them in the design process (Rogers and Fisk, 2010). The present study examined younger and older adults with three different multimedia presentation designs to determine if optimal design templates varied according to the different cognitive strengths and weaknesses of each age group. The three presentation designs were: (1) Audio only (narration), (2) Redundant text (narration with redundant text), (3) Complementary images (narration with non-redundant text and images).

Two dominant theories govern multimedia design in education: Cognitive Load Theory (CLT) proposed by Sweller et al. (1998), (Sweller, 1999, 2005), (Van Merriënboer and Sweller, 2005), and Cognitive Theory of Multimedia Learning (CTML) proposed by Mayer (2001, 2005). Both CLT and CTML build on a cognitive architecture consisting of limited capacity working memory (WM), an unlimited long-term store, and two subsystems for processing auditory and visual information (Baddeley, 1986; Cowan, 2001; Chang et al., 2011). The core features of this model include the limited capacity of WM and the independence of the subsystems (Brooks, 1968; cf. Paivio, 1986), which can simultaneously process their respective information. Purely unimodal instruction (e.g., audio narration only) does not engage these parallel processing streams, and is substantially less effective than instruction that takes advantage of both subsystems by simultaneously presenting auditory/verbal information (e.g., narration) and pictorial/non-verbal information. Critically, this multimodal presentation of information helps overcome limitations of WM.

The core features of WM models have been used to develop a myriad of multimedia design strategies to guide best practice (Mayer, 2009). For example, pairing instructional animation or images with auditory narration engages both verbal and visual processing subsystems; learners can effectively organize new information in WM and integrate this new knowledge into existing long-term memory stores, ultimately resulting in a richer memory representation. However, the impact of verbal redundancy (i.e., paring identical visual text with simultaneous narration) has been less clear. Several studies have shown that younger adults learn better when audio narration is paired with identical visual text compared to when audio narration is presented alone (Moreno and Mayer, 2002; Adesope and Nesbit, 2012). Yet others demonstrate no improvement in learning under conditions of verbal redundancy (Kalyuga et al., 2004; Fenesi and Kim, 2014; Fenesi et al., 2014). Several of these studies also show that on-screen text that is redundant with auditory narration produces substantially worse performance compared to when complementary images are paired with narration (Fenesi and Kim, 2014; Fenesi et al., 2014). Potential reasons for discrepant findings involving verbal redundancy might reflect methodological differences between studies, such as differences in material content. For example, Moreno and Mayer (2002) found a benefit of verbal redundancy for younger adults when presenting cause–effect explanations of a scientific system (e.g., lightning formation). Although this information is complex, work by Fenesi et al. (2014) presented hierarchically organized content from a subset of an actual online lecture from an introductory psychology course, where basic concepts are presented first, followed by more complex information, which builds on this foundational knowledge. Perhaps as multimedia content becomes increasingly complex and hierarchical, verbal redundancy impedes learning.

Importantly, if instructional design strategies support an efficient use of available cognitive resources, older adults should show similar patterns of learning, but might show overall reduced performance as a result of their age-related decline in WM capacity (Wingfield et al., 1988; Hedden and Gabrieli, 2004; Mattay et al., 2006; Pachman, 2007; Pachman and Ke, 2012). However, older adults may actually show enhanced performance under conditions of verbal redundancy. Indeed, older adults have demonstrated better learning with a verbally redundant presentation compared to an audio-only condition, whereas younger adults showed impaired learning under the same conditions (Pachman and Ke, 2012). In the context of driving, older adults benefited from a redundant text presentation (narration + text + map), which improved both comprehension of driving instructions and driving ability, as indexed by reduced number of lane deviations and inappropriately long glances (>2.5 s; Dingus et al., 1997). These studies suggest that presenting redundant visual text has benefits for older adult comprehension, within both multimedia and driving navigation environments.

The differential effect of verbal redundancy for older and younger participants may reflect age-related differences in cognitive function that are not fully captured by existing multimedia learning frameworks. A key aspect of cognitive aging is decreased WMC (Pachman, 2007), which reflects reduced processing resources and slower processing of incoming information. Thus, optimal learning for older adults may be promoted by reducing the reliance on internal determinants of performance (e.g., WM) and instead, relying more on external components (e.g., contextual cues, visual text) to enhance encoding and processing of presented information (Craik and Rose, 2012). Importantly, older adults show enhanced multisensory integration, especially with respect to visual dominance and the integration of visual-verbal information with auditory-verbal information (Diaconescu et al., 2012). Consequently, a redundant presentation style may enhance their learning. Note, however, that this work used low-level visuo-verbal perceptual stimuli (e.g., image of bird and chirp sound for 400 ms) to examine multisensory integration in older adults, and may not be considered scalable to high-level conceptual stimuli such as multimedia instruction. Additional evidence for the benefit of redundant presentation styles for older adults may come from attentional co-activation models (Bucur et al., 2005). According to a co-activation framework, older adults should benefit from verbal redundancy compared to younger adults, because they extract less information from the presentation of a single verbal target (e.g., narration or on-screen text alone), due to age-related reductions in WMC. Finally, narration alone or narration paired with images might lack necessary visuo-verbal support to counteract reduced auditory perception. Thus, it is possible that optimal design of multimedia for learning in older adults may be one that provides additional verbal cues in the form of redundant audio and visual text information.

The primary research objective of the current study was (1) to extend multimedia research to older adults and examine whether they show similar patterns of comprehension as younger adults, or whether they require unique multimedia design tailored to their cognitive abilities for effective learning, and (2) to replicate our prior research on younger adults that found verbal redundancy did not promote learning and that younger learners benefited most from complementary information. Unlike previous research that only examined the impact of verbally redundant text compared to audio alone (Pachman and Ke, 2012), we also examined the impact of verbally redundant text compared to complementary images; this is an important comparison as images are repeatedly shown to promote learning for younger adults, yet little is known about the impact on older adult learning. Younger and older participants were exposed to the same audio track under one of three conditions: (1) Audio only, (2) Redundant text (audio with redundant text), (3) Complementary images (audio with non-redundant text images). Both groups were then assessed for comprehension of presented material and subjective perceptions of multimedia quality and effectiveness.

According to prior research, (Kalyuga et al., 2004; Fenesi and Kim, 2014; Fenesi et al., 2014), younger participants were expected to have better comprehension performance when the audio track was presented with complementary images compared to redundant text or audio only. This is based on the theoretical assumption that redundant verbal information overwhelms the auditory/verbal subsystem and reduces critical WM resources needed to meaningfully understand and integrate incoming information. Presenting narration and relevant images allows visual/pictorial and auditory/verbal subsystems to function in parallel, promoting optimal WM resource allocation. For older participants, if we assume that existing instructional theories support an efficient use of available cognitive resources and are equally beneficial across age groups, we would predict a similar pattern of results for both younger and older adults, with both age groups performing best in the Complementary condition and worst in the Redundant text and Audio conditions. In contrast, if we assume that older adults cannot rely as effectively as younger adults on internal determinants of performance (i.e., WMC and processing resources), they may benefit from redundant text due to greater verbal ability, attentional co-activation or visual text functioning as an external contextual aid; in this case, we would predict older adults would show a different pattern of results from younger adults and perform best in the Redundant text condition compared to the Complementary and Audio conditions.

The secondary research objective was to examine how subjective perceptions of the multimedia presentation interacted with age group and presentation design, and if these subjective factors influenced comprehension. Prior work has demonstrated discrepancies between objective and subjective measures of comprehension, with younger adults believing ineffective presentations aid their understanding (Fenesi and Kim, 2014; Fenesi et al., 2014). Similarly, older adults also show limited accuracy in judging the effectiveness of learning strategies, rating rote memorization as an effective learning strategy, even though it is not (Hertzog and Dunlosky, 2004). We wanted to extend this research to judgments of multimedia design quality and effectiveness in older adults, and evaluate whether both younger and older adults have equally poor judgments of effective instructional design.

## Materials and Methods

### Participants

#### Young Adults

**Table 1** provides demographic information for both age groups. One hundred and one first year undergraduate students from McMaster University, 27 men and 64 women (*M* age = 18.75, SD = 2.12) participated in the experiment and were randomly assigned to one of three conditions: Audio (*M* age = 18.86, SD = 2.5, *N* = 35), Redundant (*M* age = 18.33, SD = 1.02, *N* = 33), and Complementary (*M* age = 19.06, SD = 3.38, *N* = 33). All participants were enrolled in Introductory Psychology and received course credit. They were recruited using an online portal designed for psychology research. Participants were prescreened to ensure they had not taken previous anatomy courses, or been previously exposed to content related to hunger mechanisms in their Introductory Psychology course. All participants provided informed consent, and all procedures complied with the tri-council statement on ethics, as assessed by the McMaster Research Ethics Board.

**Table 1:** Demographic information across both younger and older adults for age, sex, total years of education, number of hours spent on a computer per week, and total online courses taken in a lifetime.

	Younger			Older		
	Audio *M* (SD)	Redundant *M* (SD)	Complementary *M* (SD)	Audio *M* (SD)	Redundant *M* (SD)	Complementary *M* (SD)
N	34	33	33	27	24	24
Age	18.85 (2.50)	18.33 (1.02)	19.06 (3.38)	71.63 (5.26)	73.08 (5.82)	72.04 (5.20)
Sex	*f* = 22	*f* = 22	*f* = 29	*f* = 20	*f* = 20	*f* = 17
Education (yrs)	13.69 (1.28)	13.23 (1.13)	13.94 (1.41)	16.63 (4.81)	17.04 (2.58)	17.65 (3.92)
**Computer use/week (hrs)**						
0	0	0	0	2	2	1
<1	0	0	0	3	1	0
1–3	0	1	0	4	1	3
4–6	3	2	1	11	3	4
7–10	2	7	5	2	2	8
11–15	6	8	4	1	7	3
16–20	12	4	10	1	1	3
20+	11	11	13	3	7	2
Total online courses	2.29 (1.69)	2.42 (2.05)	2.55 (1.09)	1.48 (3.25)	0.95 (1.94)	2.46 (3.54)

#### Older Adults

Seventy-five older participants from the Baycrest Research Subject Pool, 25 men and 50 women (*M* age = 72.36, SD = 5.31) were recruited via telephone interview based on the following inclusion criteria: healthy volunteers, age over 65, fluent in English, functional hearing and vision, no major neurologic illness, no current untreated psychiatric or substance-related disorder, and no severe sensory impairment (normal or corrected to normal hearing and vision). Participants received $10 monetary compensation. They were randomly assigned to one of three conditions: Audio (*M* age = 71.63, SD = 5.26, *N* = 27), Redundant (*M* age = 73.08, SD = 5.82, *N* = 24), and Complementary (*M* age = 72.04, SD = 5.2, *N* = 24). All participants provided informed consent, and all procedures complied with the Baycrest Human Subjects Research Ethics Board.

### Stimuli and Procedure

Participants were randomly assigned to view one of three multimedia presentations: (1) Audio only, (2) Redundant text (audio narration paired with redundant on-screen text), or (3) Complementary (audio narration paired with images and minimal text). Appendix A (Supplementary Material) provides example slides for the Redundant and Complementary conditions (Audio condition was a blank screen), along with web links to view the actual presentations. Each presentation consisted of a 9-min system-paced PowerPoint slide show (total of 23 slides) about the physiology, anatomy, evolution, and biochemical mechanisms of hunger. The narration was 1375 words (80 sentences), and was rated as requiring an 11.02 grade-school level of reading skill to effectively read the text (using Flesch–Kincaid Grade Level formula; Farr et al., 1951). The narration was also rated as having an average ease of readability using the Flesch Reading Ease inventory (scored 46.67 which falls within the range considered average of 6–70).

The Redundant condition consisted of 2–4 bullet points of text (Calibri font, size varied between 20 and 24) per slide (verbatim to the slide’s narration). The location of the text was always within 1 inch left–right margins, and 0.5–1 inch top–bottom margins of the screen, with minor deviations due to slightly different amounts of text across slides. Text size and density varied slightly across slides to ensure both Redundant and Complementary conditions consisted of 23 slides total, and that each slide across both conditions presented the same amount of content. The loudness of the audio narration was adjusted for each participant, since they listened to the presentation narration via individual headsets. The experimenter presented a non-experimental video with sound prior to the beginning of the presentation, and allowed each participant to adjust the volume to a comfortable, audible level. Participants were also shown the volume control keys so that they could adjust the volume at any time during the experiment. At the end of the experiment, all participants were probed for audibility, with no participants reporting difficulty hearing. Additionally, all participants were prescreened for sufficient hearing; older adults that indicated hearing deficiencies had hearing aids, and indicated no hearing difficulties during the experiment.

Within the Complementary condition, the size of images varied depending on slide content (e.g., image of gastrointestinal tract was larger and more visually dense than an image of a balance beam with glycogen and glucose on opposite sides depicting the hunger process). The duration of each slide across conditions varied depending on the slide’s content. Some slides were more content-heavy, requiring longer presentation durations of text and images (although slide duration was identical across conditions).

Younger participants individually viewed a multimedia presentation on a 15-inch Dell laptop with an attached headset. Older participants viewed the presentation on individual Dell desktop PCs with 19-inch displays and an attached headset. For both age groups, the experiment took 40–60 min to complete (5-min instructions, 9 min presentation, 30–40 min comprehension quiz and questionnaire, 5-min debrief). There were 5–8 participants in each session, each on their own individual computer. Immediately after viewing the presentation, participants responded to the comprehension quiz, followed by a perception and technology use questionnaire.

Comprehension performance was determined by participants’ mean score on 20 multiple-choice questions (four-option answers). Two different question types were used to diversify the questions: 10 questions evaluated basic retention and 10 questions evaluated problem-transfer (see Appendix B for the complete comprehension quiz—Supplementary Material). An online survey system (LimeSurvey) was used to collect all data.

Perception measures were assessed by the participant’s response to four statements: (1) I found the material presented in this lecture to be interesting (interest), (2) The lecture material has a high level of difficulty (difficulty), (3) I found the multimedia presentation to be engaging (engagement), and (4) I found that the presentation style helped me to understand the lecture material (understanding). Response options to all perception measures were reported on a four-point scale (1 = absolutely disagree, 2 = mostly disagree, 3 = mostly agree, 4 = absolutely agree). Importantly, each perception measure was associated with a specific feature of the multimedia presentation. That is, perception measures of interest and difficulty required participants to reflect on the content of the presentation (i.e., lecture information), whereas perception measures of engagement and understanding required participants to reflect on the actual presentation design (i.e., use of words and images). Previous research has strongly encouraged the collection of both perception measures and performance indicators (i.e., comprehension) to better represent product quality (Moullin, 2004).

A computer-use measure was also included to determine whether time spent using a computer was related to comprehension performance or subjective perception measures. This was used to establish whether experimental conditions were equal with respect to computer-related technical prerequisites. Participants responded to the statement: What is the *total* number of hours a week that you spend on a computer? Response options were: 0, less than 1 h, 1–3 h, 4–6 h, 7–10 h, 11–15 h, 16–20 h, 20+ h. Additionally, participants were asked to indicate how many online courses they had taken in their lifetime. All participants were debriefed following the experiment.

### Analysis

Comprehension scores and perception measures were analyzed using a 2 (age group: young, old) × 3 (presentation condition: Audio, Redundant, Complementary) factorial ANOVA. Alpha was set to 0.05 for all main effects and interactions, and all pairwise comparisons using independent samples *t*-tests were Bonferroni corrected. Effect sizes were calculated for main effects, interactions, simple main effects and pairwise comparisons (cohen’s *d* was used for independent *t*-tests, and partial eta squared, $\eta_{p}^{2}$, was used for ANOVA). Two correlation matrixes (one for each age group) were also used to assess the relation between technology use and dependent measures of comprehension performance and subjective perception ratings. SPSS 20 for Macintosh was used to conduct data analyses.

## Results

For both age groups, there were no significant correlations between technology use and comprehension, and no significant correlations between technology use and perception measures (all *r*s < 0.2). Therefore, the amount of computer-use across age groups was not related to comprehension performance or subjective perception measures. There were no significant differences in years of education or number of online courses taken within a lifetime across the three conditions for both age groups, as indicated by non-significant one-way ANOVAs (all *F*s < 2.63, *p* > 0.07). These analyses indicate effective random assignment across conditions within both age groups; all conditions within an age group consisted of participants with similar years of education and online educational exposure. As expected, older adults also had significantly more years of education than younger adults, as indicated by an independent samples *t*-test, *t*(86) = 7.42, *p* < 0.001, *d* = 1.2.

### Comprehension Performance

**FIGURE 1:**
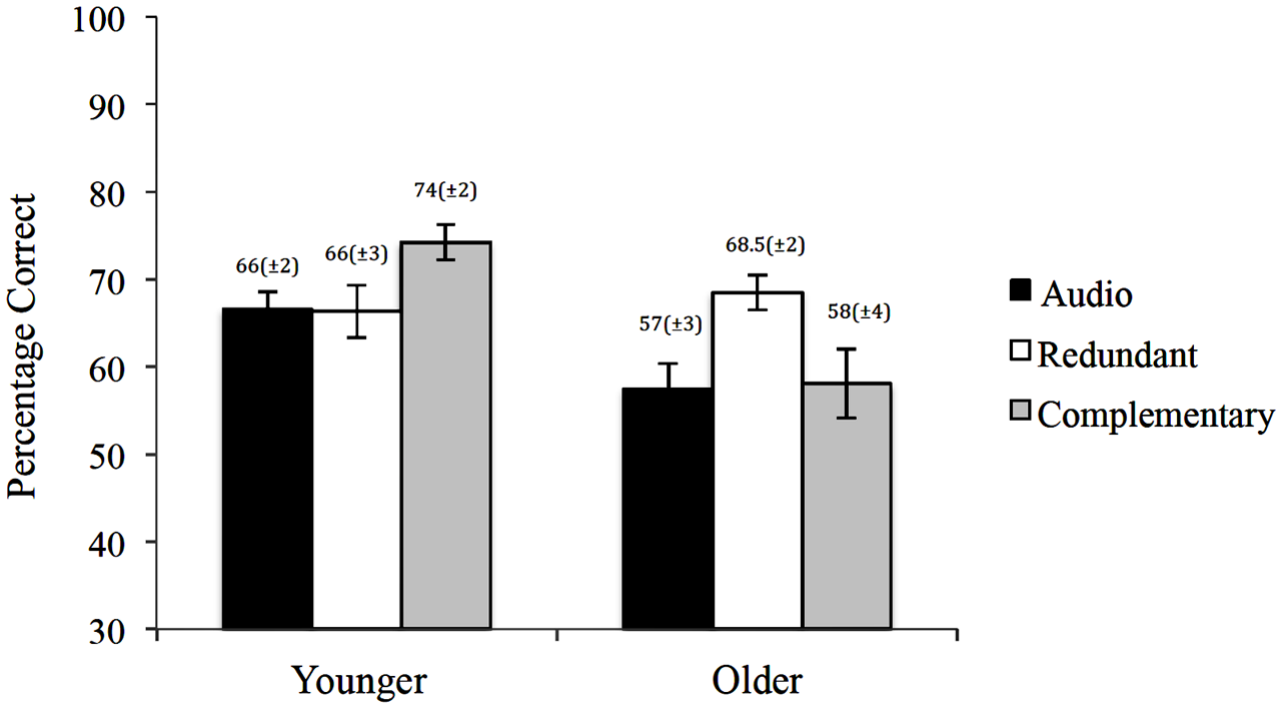
**Differences in comprehension performance between presentation conditions for both younger and older adults (bars represent SE)**.

Comprehension scores are presented in **Figure 1**. Preliminary analyses found no differences among conditions (across both age groups) in comprehension scores between basic retention and problem-transfer questions; we therefore collapsed across question type. Younger participants had higher comprehension scores than older participants, supported by a main effect of age, *F*(1,170) = 13.37, *MSE* = 0.19, *p* < 0.001, $\eta_{p}^{2}$ = 0.07. Comprehension scores among the three conditions was similar when collapsing across the age groups, as indicated by a non-significant main effect of condition *F*(2,170) = 2.53, *MSE* = 0.19, *p* = 0.08., $\eta_{p}^{2}$ = 0.03. The presentation condition had differential effects on the two age groups, *F*(2,170) = 6.22, *MSE* = 0.19, *p* = 0.002, $\eta_{p}^{2}$ = 0.07. For the younger participants, the Complementary condition had the best comprehension performance, which replicates previous findings (Fenesi et al., 2014). There was no difference between the Redundant and Audio conditions [*t*(66) = 0.06, *p* = 0.95], which were both worse than the Complementary condition [*t*(64) = –2.53, *p* = 0.01, *d =* 0.64 and *t*(66) = –2.87, *p* = 0.01, *d =* 0.35, respectively]. In contrast, older participants in the Redundant condition had the best performance. There was no difference between the Complementary and Audio conditions [*t*(64) = –0.15, *p* = 0.89], which were both worse than the Redundant condition [*t*(46) = 2.51, *p* = 0.02, *d =* 0.76 and *t*(49) = –2.8, *p* = 0.01, *d =* 0.83, respectively].

### Subjective Perception

#### Perceived Understanding

**FIGURE 2:**
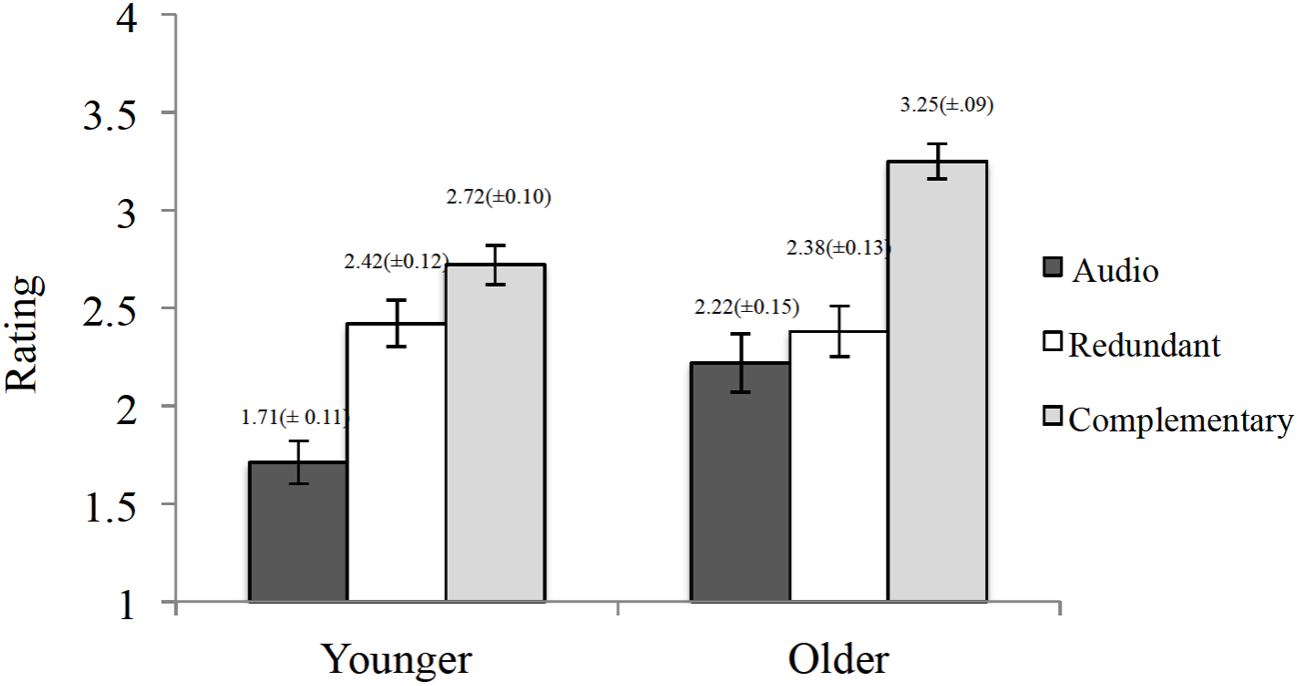
**Differences in perceived understanding ratings between presentation conditions for both younger and older adults (bars represent SE)**.

**Figure 2** shows perceived understanding ratings among conditions for both age groups. Older adults had higher perceived understanding ratings than younger adults, which was supported by a main effect of age for understanding *F*(1,170) = 11.08, *MSE* = 0.42, *p* = 0.001, $\eta_{p}^{2}$ = 0.06. When collapsing across age groups, ratings of perceived understanding differed across conditions (main effect of condition: *F*(2,170) = 36.62, *MSE* = 0.42, *p* < 0.001, $\eta_{p}^{2}$ = 0.30) and this interacted with age (age × condition interaction: *F*(2,170) = 3.61, *MSE* = 0.42, *p* = 0.03, $\eta_{p}^{2}$ = 0.04). Younger adults in the Complementary and Redundant conditions had higher ratings of perceived understanding than those in the Audio condition [Redundant vs. Audio, *t*(66) = –4.4, *p* < 0.001, *d =* 1.06; Complementary vs. Audio, *t*(66) = –6.69, *p* < 0.001, *d =* 1.61]. In contrast, older adults in the Complementary condition had higher ratings of perceived understanding than the Redundant or the Audio conditions [Complementary vs. Redundant, *t*(46) = –5.47, *p* < 0.001, *d =* 1.58; Complementary vs. Audio, *t*(49) = –5.57, *p* < 0.001, *d =* 1.66]. Spearman correlations revealed no significant relation between subjective and objective measures of understanding for both age groups (*r* < 0.146).

#### Perceived Interest, Engagement, and Difficulty

**Table 2** shows perception ratings among age groups and conditions. Differences across conditions were observed for ratings of *interest* [*F*(2,170) = 5.44, *MSE* = 0.49, *p* = 0.01, $\eta_{p}^{2}$ = 0.06] and *engagement* [*F*(2,170) = 26.22, *MSE* = 0.52, *p* < 0.001, $\eta_{p}^{2}$ = 0.24], but not *difficulty* [*F*(2,170) = 0.44, *MSE* = 0.44, *p* = 0.65, $\eta_{p}^{2}$ = 0.01]. Older adults rated the presentations as more interesting, engaging, and difficult than younger adults [main effect of age for *interest: F*(1,170) = 6.98, *MSE* = 0.49, *p* = 0.01, $\eta_{p}^{2}$ = 0.04, *engagement: F*(1,170) = 39.03, *MSE* = 0.52, *p* < 0.001, $\eta_{p}^{2}$ = 0.19, and *difficulty: F*(1,170) = 38.59, *MSE* = 0.44, *p* < 0.001, $\eta_{p}^{2}$ = 0.19]. Younger adults ratings of interest did not differ between conditions (all *t*s < 1.44). In contrast, older adults in the Complementary condition had higher ratings of interest than older adults in the Redundant and Audio conditions as indicated by a significant interaction [*F*(2,170) = 5.49, *MSE* = 0.49, *p* = 0.01, $\eta_{p}^{2}$ = 0.06], and pairwise comparisons [Complementary vs. Redundant: *t*(46) = –2.67, *p* = 0.01, *d =* 0.78; Complementary vs. Audio: *t*(49) = –4.12, *p* < 0.001, *d =* 1.23]. With respect to engagement, the presentation condition did not have differential effects on the two age groups, as indicated by a non-significant interaction *F*(2,170) = 2.65, *MSE* = 0.19, *p* = 0.07, $\eta_{p}^{2}$ = 0.03. Looking more closely, younger adults in the Redundant and Complementary conditions had higher ratings of engagement than younger adults in the Audio condition [Redundant vs. Audio, *t*(66) = –2.84, *p* = 0.01, *d =* 0.69; Complementary vs. Audio, *t*(66) = –5.18, *p* < 0.001, *d =* 1.26], while older adults in the Redundant condition had equivalent ratings to older adults in the Audio condition [*t*(49) = –0.05, *p* = 0.96], and the older adults in the Complementary condition had higher ratings of engagement than the Redundant condition [*t*(46) = –5.1, *p* < 0.001, *d =* 1.48]. Additionally, both younger and older adults rated the conditions as being of equal difficulty [all *t*s < 1.44, non-significant interaction *F*(2,170) = 1.41, *MSE* = 0.44 *p* = 0.06, $\eta_{p}^{2}$ = 0.02].

**Table 2:** Mean ratings of perceived presentation material difficulty, engagement, and interest for both age groups (±SE).

	Younger			Older		
	Audio	Redundant	Complementary	Audio	Redundant	Complementary
Difficulty	2.71 ± 0.12	2.42 ± 0.11	2.46 ± 0.11	3.11 ± 0.14	3.21 ± 0.10	3.17 ± 0.16
Engagement	1.57 ± 0.12	2.09 ± 0.14	3.38 ± 0.12	2.41 ± 0.13	2.42 ± 0.15	3.38 ± 0.12
Interest	1.71 ± 0.11	2.42 ± 0.12	2.73 ± 0.10	2.78 ± 0.17	3.21 ± 0.12	3.63 ± 0.10

## Discussion

Two main results emerge from the data. First, the replication of superior comprehension for complementary images observed for younger learners was not seen in the older learners. Instead, older learners performed better with redundant text. Second, there was a lack of metacognitive awareness as indicated by a non-significant relation between subjective and objective measures of comprehension, with both groups rating a non-optimal condition for their age group as more effective in learning. These results highlight the importance of considering age-related differences in learning, and the poor awareness learners have of their, respectively, effective multimedia presentation design.

In line with previous findings (Fenesi and Kim, 2014; Fenesi et al., 2014), younger adults benefited most from a Complementary presentation, where pictorial and verbal information were simultaneously presented in separate processing streams; according to CLT and CTML, this may have helped younger adults maximize WM resource allocation by promoting visual/pictorial and auditory/verbal subsystems to function in parallel (Sweller, 1999; Mayer, 2001). This presentation is also believed to facilitate the construction of mental representations of information, which helps consolidate new information with pre-existing knowledge. However, presenting redundant on-screen text may have overwhelmed their auditory/verbal processing subsystem and reduced critical WM resources needed to meaningfully understand and integrate incoming information.

In contrast, older adults learned better with redundant text than images. These findings add to the existing research that demonstrates older adults have superior comprehension of information with redundant text compared to audio only (Dingus et al., 1997; Pachman and Ke, 2012). Pairing on-screen text with narration provides external contextual aid and may help reduce reliance on cognitive determinants of ability (i.e., WM), thereby enriching the perceptual detail of the presentation and enhancing older adult learning. Additionally, older adults might have difficulty attending to relevant words in the narration (known as selecting in CTML), so providing on-screen text helps compensate for insufficiently selected auditory-verbal input (Mayer, 2009).

These findings also suggest that older adults may have superior multisensory integration not only during exposure to low-level perceptual stimuli (Diaconescu et al., 2012), but also during exposure to high-level conceptual stimuli such as multimedia instruction; older adults are likely better able to integrate visual-verbal information with auditory-verbal information during multimedia learning, resulting in enhanced learning with redundant verbal information. These results also support an attentional co-activation framework, as older adults were likely less effective at extracting information from a single verbal target (e.g., narration alone), due to age-related reductions in WMC (Bucur et al., 2005). Thus, the presentation of an additional redundant target (i.e., on-screen text) promoted comprehension. Furthermore, given that older adults had significantly more years of education, which is strongly linked to greater verbal ability (Steffener et al., 2014), they may have been more efficient at encoding verbal information without overwhelming WMC, leading to superior performance when exposed to redundant verbal information (Lien et al., 2006). However, in order to fully establish a model of redundant coding across modalities for older adults, future work should include a text-only condition (without narration) to directly test the contributions of redundant coding versus text-only for older adults learners. The current study did not include a text-only condition because the primary objective was to evaluate whether instructional design that promotes learning for younger adults yields similar benefits for older adults. Furthermore, all presentations were system-paced to ensure participants received the same rate of instruction. This eliminated potential confounds associated with individual differences in reading speed, variance in time-on-task behavior, and potential re-exposure to context through re-reading of information.

Lower performance in the Complementary condition for older adults might reflect their reduced capacity to engage in deep processing (known as integrating in CTML), which is required to effectively integrate words and images (Mayer, 2009). Another explanation could reflect age-related cognitive declines in the ability to coordinate complex information (Van Gerven et al., 2006). The Complementary condition could pose a coordination complexity, since it may have required processing and integrating multiple sources of information (i.e., verbal and pictorial). Therefore, although providing images should help externalize some of the instructional demand (by providing pictorial aid), the requirement to regulate and monitor information between processing steps may have created a learning task too high in complexity. Importantly, our conclusions are based on well-established theoretical frameworks of multimedia design (i.e., CLT and CTML), which are heavily entrenched in an understanding of WM processes and limitations. Also, there is extensive aging research demonstrating a robust pattern of reduced WMC as a function of increasing age (Paas et al., 2001; Hedden and Gabrieli, 2004; Van Gerven et al., 2006). Thus, our findings that older adults learn differently from multimedia instruction due to age-related reductions in WMC are well supported by extensive research in cognitive aging.

With respect to subjective perceptions, the results reveal poor metacognitive awareness across the lifespan. Younger adults in the Redundant condition falsely perceived this template to facilitate understanding. These results replicate prior findings (Fenesi et al., 2014) and further highlight the inaccurate value young learners place on the use of redundant text. Although the present study was a between-subjects design, and participants were only exposed to one of the three conditions and therefore could not directly compare different presentation styles, other studies using a within-subjects design have supported the claim that younger adults prefer redundant text despite direct exposure to more effective presentation styles within the same experimental session (Fenesi and Kim, 2014). Students may view redundant text as a positive learning tool due to its common use within classrooms (Pina and Savenye, 1992). Instructors often use redundant text to conveniently organize and execute required lesson plans, but may not realize that such presentations do not improve comprehension. As a result of repeated educational exposure, learners may develop a sense of familiarity and comfort with such presentations (Hansen and Wanke, 2009), driving the belief that redundant text promotes learning.

Older adults showed an even greater lack of awareness for multimedia presentations that helped or hindered their learning; older adults in the Complementary condition had higher ratings of interest, engagement, and understanding compared to older adults in the Redundant condition. Perhaps older learners are less exposed to the text-heavy PowerPoint culture that accompanies many university lectures. They therefore do not have a sense of familiarity and comfort with such presentations, which may reduce any familiarity-driven preference for redundant text. Older adults may also fail to recognize on-screen text as an external contextual aid, even though their cognitive processing mechanisms rely on the additional environmental support to enhance comprehension. However, it is unclear whether older adults will subjectively prefer a Complementary presentation style when they are able to directly contrast it with a Redundant presentation style. Future work should employ a within-subjects design to test this question (c.f., Fenesi and Kim, 2014).

This study reinforces the need for multimedia design research to continue investigating differential advantages of prescribed multimedia design strategies across younger and older adults. Effective multimedia design for older adults is especially important considering they typically have lower technology-related self-efficacy and higher computer anxiety than younger adults (Czaja and Sharit, 1998; Czaja et al., 2006). Although there may be baseline cohort differences between younger and older adults in technology exposure and efficacy, well-designed multimedia has the potential to help older adults become more comfortable with instructional technology and promote learning. While there may be many design principles that benefit both age groups, this study demonstrates that effective multimedia design can vary depending on learner age. With an educational culture that is increasingly technology-based, it is important for instructional design research to be inclusive of diverse age groups.

## Conflict of Interest Statement

The authors declare that the research was conducted in the absence of any commercial or financial relationships that could be construed as a potential conflict of interest.
