# Supplementary material for: One size does not fit all: older adults benefit from redundant text in multimedia instruction
Source: Front Psychol. 2015 Jul 28;6:1076. doi: 10.3389/fpsyg.2015.01076 (PMC4516814; doi:10.3389/fpsyg.2015.01076)
Supplement: Supplementary file 1 [file Presentation_1.PDF]

Appendix A

Example slide from Redundant and Complementary presentation conditions

Audio only (*http://bit.ly/1rJJMvG)*

Redundant Presentation (*http://bit.ly/1pjYJN8)*

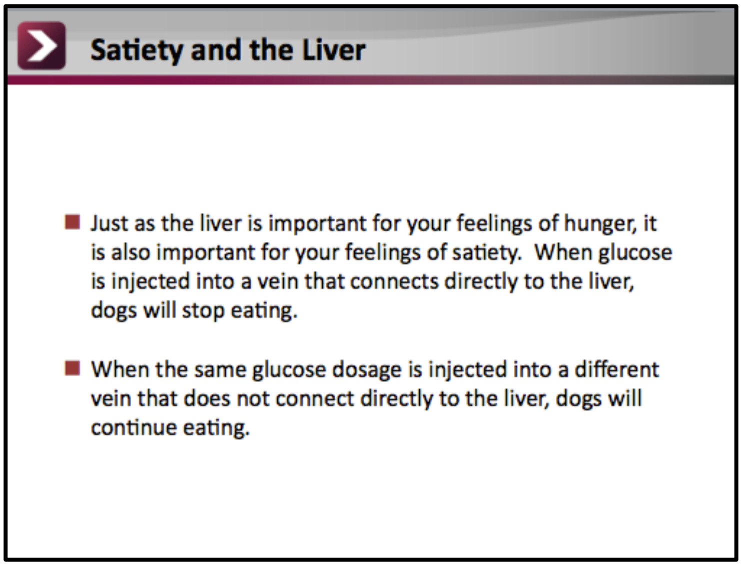

Complementary Condition (*http://bit.ly/1A0Mewc)*

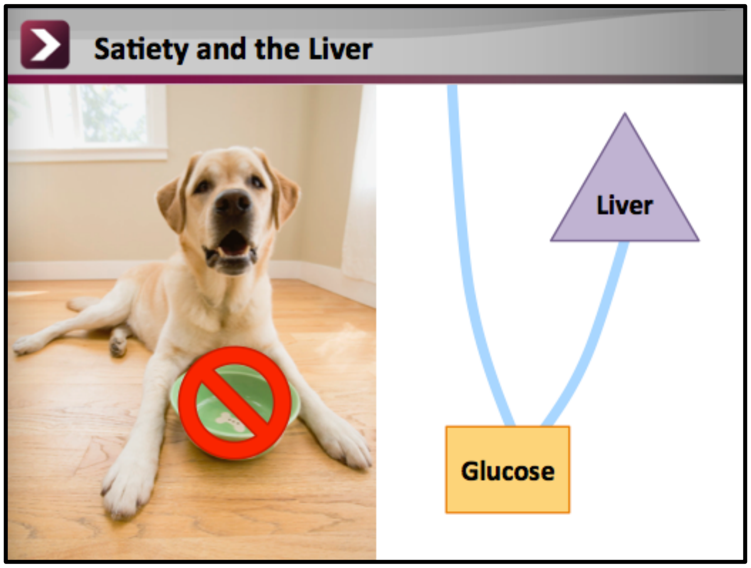

Appendix B

Comprehension quiz (answers are bolded)

Recognition Comprehension Questions

In 1944, Inglefinger studied cancer patients with their stomachs surgically removed. Describe what his study concluded about feelings of hunger?

a. You need a stomach in order to feel hungry
b. You do not need a stomach in order to feel hungry
c. Individuals without stomachs do not report feelings of hunger
d. You need only part of your stomach to feel hungry

Physiological evidence indicates that part of the _____ controls the cessation of feeding. It appears to do so by ______.

a. hypothalamus…monitoring stomach distension
b. thalamus…monitoring the rate of glucose use
c. hypothalamus…monitoring the rate of glucose use
d. limbic system…monitoring the rate of glucose use

As glucose levels drop:

a. You start feeling full
b. Remaining glucose is quickly converted into glycogen
c. Glycogen is broken down into glucose
d. Fat is stored

Which nutrient signals the need to replenish one's food intake?

a. glucose
b. fructose
c. adipose tissue
d. glycogen

What hormone, produced in the small intestine, is hypothesized to be related to feelings of fullness?

a. PYY
b. CCK
c. Leptin
d. Glucose

According to the lecture, which part of the brain is the most important in the regulation of hunger and satiety?

a. olfactory bulb
b. prefrontal cortex

c. hippocampus
d. hypothalamus

According to the lecture, "our lives seem dominated by the consumption of food". What was the evolutionary rationale behind this statement?

a. In the past, humans had to expend more effort in order to find food than is typical for modern industrial societies today.
b. In the past, humans had to expend minimal effort in order to find than is typical for modern industrial societies today.
c. In the past and in present industrial societies, humans expend a great deal of energy seeking out scarcely available food.
d. In the past and in present industrial societies, humans expend less energy seeking out scarcely available food.

Stimulation of the ventromedial nucleus of the hypothalamus in rats might be expected to cause:

a. an increase in food intake and weight gain
b. a sharp decrease in food intake (or its complete cessation) and weight loss
c. a transition from waking to sleep, if the stimulation is of high frequency
d. permanent wakefulness

Damage to the brain area important in regulating eating behaviour can affect hunger and satiety in two different ways. What are they?

a. overeating only
b. refusing to eat only
c. overeating, or refusing to eat
d. emotional overeating only

What is the role of adipose tissue?

a. stores energy for later use
b. signals the body to replenish its food intake
c. maintains the body at a healthy weight
d. carries glucose to different areas of the body

<u>*Problem–Transfer Comprehension Questions*</u>

A recent visit to the doctor reveals your large intestine is not secreting the PYY hormone. Choose the most likely consequence you would encounter?

a. You may not be able to feel full
b. You may not be able to feel hungry
c. Your liver will be unable to convert glycogen to glucose

d. You may feel constantly full

Dr. Burn is testing the role of the liver in monitoring glucose levels to control feeding behaviour. Although he injects two similar dogs with a sufficient load of glucose, one stops eating while the other continues to eat. Solve what is most likely to have happened?

a. The dog that continues to eat is extremely hungry and the glucose had no impact
b. The dog that continues to eat is low on glycogen levels
c. The dog that continues to eat had the glucose injected into a vein that does not reach the liver
d. The dog that continues to eat had the glucose injected into a vein that does reach the liver

You have discovered an animal that does not seem to employ glycogen stores (or an equivalent). Applying your knowledge about glycogen stores, you might expect this animal to:

a. Eat frequently and have highly variable glucose levels
b. Eat frequently and have consistently low glucose levels
c. Eat infrequently and have highly variable glucose levels
d. Eat infrequently and have consistently low glucose levels

Dr. Smith discovers one of his patients (Mike) has been gaining weight. Upon closer inspection, Dr. Smith discovers Mike's leptin levels are abnormally low. What role does leptin play in long-term weight regulation?

a. When fat tissue increases, leptin production is halted, and daily food consumption is lowered
b. When an individual feels hungry, leptin levels rise, and signal the body to consume food
c. When an individual feels hungry, leptin levels rise, and signal the body to reduce food consumption
d. When fat tissue increases, leptin levels rise, and is involved in reducing daily food consumption.

Peter's liver is correctly identifying glucose levels in his blood. Frank's liver is incorrectly identifying glucose levels in his blood. What would be the difference between Peter and Frank's liver activity?

a. Peter's but not Frank's liver would be breaking down glycogen into glucose when glucose levels are low
b. Frank's but not Peter's liver would be breaking down glycogen into glucose when glucose levels are low
c. Peter's but not Frank's liver would be converting glucose into glycogen when glycogen levels are low
d. Frank's but not Peter's liver would be converting glucose into adipose tissue when glycogen levels are low

A pharmaceutical company is trying to create a drug that will help obese clients lose weight. Taking advantage of what you have learned thus far, which of the following approaches would be best?

a. Create a drug that mimics the function of Leptin

b. Create a drug that stimulates the liver to break down glycogen to glucose
c. Create a drug that blocks the receptors of NPY
d. Create a drug that stimulates the overproduction of adipose tissues

Dr. Smith discovers the presence of a hormone in the small intestine, which he hypothesizes, causes feelings of fullness, and reduces eating. Which following observation (if found) would argue against his hypothesis?

a. When this hormone was injected into subjects, it resulted in feelings of nausea. Therefore, perhaps nausea, and not a feeling of fullness, reduced food consumption.
b. When this hormone was injected into subjects, it caused stomach constriction and intense gastrointestinal pain. Therefore, perhaps pain, and not a feeling of fullness, reduced food consumption.
c. When this hormone was injected into subjects, it caused the esophagus to constrict, consequently preventing food consumption. Therefore, perhaps esophagus constriction, and not a feeling of fullness, reduced food consumption
d. When this hormone was injected into subjects, feelings of fatigue resulted. Therefore, perhaps fatigue, and not feelings of fullness, reduced food consumption.

In a transporter malfunction, John's stomach was accidently removed. What effect will it have on his eating habits?

a. John will eat more
b. John will eat slightly less
c. John will now experience highly variable levels of hunger
d. No effect

John acquires a head injury during a car accident and over the subsequent weeks he gains over 80 pounds. What may have been the cause for his excessive weight gain?

a. Overproduction of CCK in the brain
b. Lateral hypothalamus damage
c. Ventromedial hypothalamus damage
d. Damage to hypothalamus inhibiting production and release of NPY

Dr. Burn has discovered a new hormone called DBH that he believes directly inhibits the actions of NPY. Which of the following experimental procedures would allow Dr. Burn to test his hypothesis?

a. Inject DBH into the hypothalamus; if eating increases, his hypothesis is correct
b. Inject DBH into the hypothalamus; if eating decreases, his hypothesis is correct
c. Inject DBH into the liver; if eating increases, his hypothesis is correct
d. Inject DBH into the liver, if eating decreases, his hypothesis is correct
